# Challenges and Opportunities for Public Health Service in Oman From the COVID-19 Pandemic: Learning Lessons for a Better Future

**DOI:** 10.3389/fpubh.2021.770946

**Published:** 2021-12-09

**Authors:** Sulien Al Khalili, Amal Al Maani, Adil Al Wahaibi, Fatma Al Yaquobi, Amina Al-Jardani, Khalid Al Harthi, Abdullah Alqayoudhi, Abdullah Al Manji, Bader Al Rawahi, Seif Al-Abri

**Affiliations:** Directorate General for Disease Surveillance and Control, Ministry of Health, Muscat, Oman

**Keywords:** COVID-19, Oman, public health, disease surveillance, One Health, health care system, pandemic

## Abstract

Despite the apparent challenges inflicted by COVID-19 globally, the pandemic provided an opportunity to utilize and expand existing public health capacities for a more adaptive and resilient system during and after each wave of the disease. This paper provides a narrative review of Oman's public health response to the COVID-19 pandemic from January 2020 to July 2021, and the challenges it faced for a more rapid and efficient response. The review demonstrates that the three main pillars influencing the direction of the pandemic and aiding the control are Oman's unified governmental leadership, the move to expand the capacity of the health care system at all levels, and community partnership in all stages of the response including the COVID-19 vaccination campaign. The opportunities identified during response stages in the harmonization of the multisectoral response, streamlining communication channels, addressing vulnerable communities (dormitories, residences at border regions), and providing professional technical leadership provide an excellent precursor for expediting the transformation of Oman's health care system to one with a multisectoral holistic approach. Some of the major challenges faced are the shortage of the public health cadre, lack of a fully integrated digital platform for surveillance, and the scarcity of experts in risk communication and community engagement. A future health system where the center for diseases surveillance and control acts as a nucleus for multisectoral expertise and leadership, which includes community representatives, is crucial to attain optimum health. The destruction inflicted by this prolong COVID-19 pandemic at all levels of human life had valued the importance of investing on preventive and preparedness strategies.

## Introduction

The unprecedented events of the COVID-19 pandemic have placed enormous strain on public health systems worldwide, exposing numerous opportunities for improvement and future preparedness. The ongoing impact of the COVID-19 pandemic since the World Health Organization first declared it emphasizes the need to change, in terms of a pandemic combating strategy, from unidirectional short-term solutions toward a holistic, integrated, and multisectoral approach (1).

Experts in global health have been advocating for a “syndemic” or synergistic epidemic approach to the management of communicable and non-communicable diseases, recognizing that diseases occur alongside social and ecological conditions (2–5). Looking at COVID-19 from a syndemic perspective through multisectoral approach may add more value to individuals and communities.

Oman is a Middle Eastern country with a population of 4.6 million of which 41% are migrants.^1^ The country has made efforts to meet the needs of International Health Regulations (2005) (IHR) (6). In April 2017, Oman underwent a Joint External Evaluation (JEE) of the IHR core capacities which included a measurement of public health capacities. The JEE found that the collaboration between different response sectors is a strength for Oman (7). However, despite magnificent effort and steps made in the field of public health in Oman recently, the pandemic revealed that the country was fit to make it through a short event but unprepared for a pandemic of the magnitude of COVID-19.

The current article describes COVID-19 pandemic public health management in Oman from January 2020 to July 2021. During this time, Oman faced multiple unique challenges including transition to a new government, financial crises, presence of multiple borders, and socio-economic connections with neighboring countries, large extended families and the dormitories of migrant workers, as well as challenges within the healthcare system. This review addresses how the COVID-19 pandemic has generated opportunities that position Oman to make steps to align their various efforts in health care adding value and minimizing destruction in future similar situation and the ongoing public health care services.

**Figure 1:**
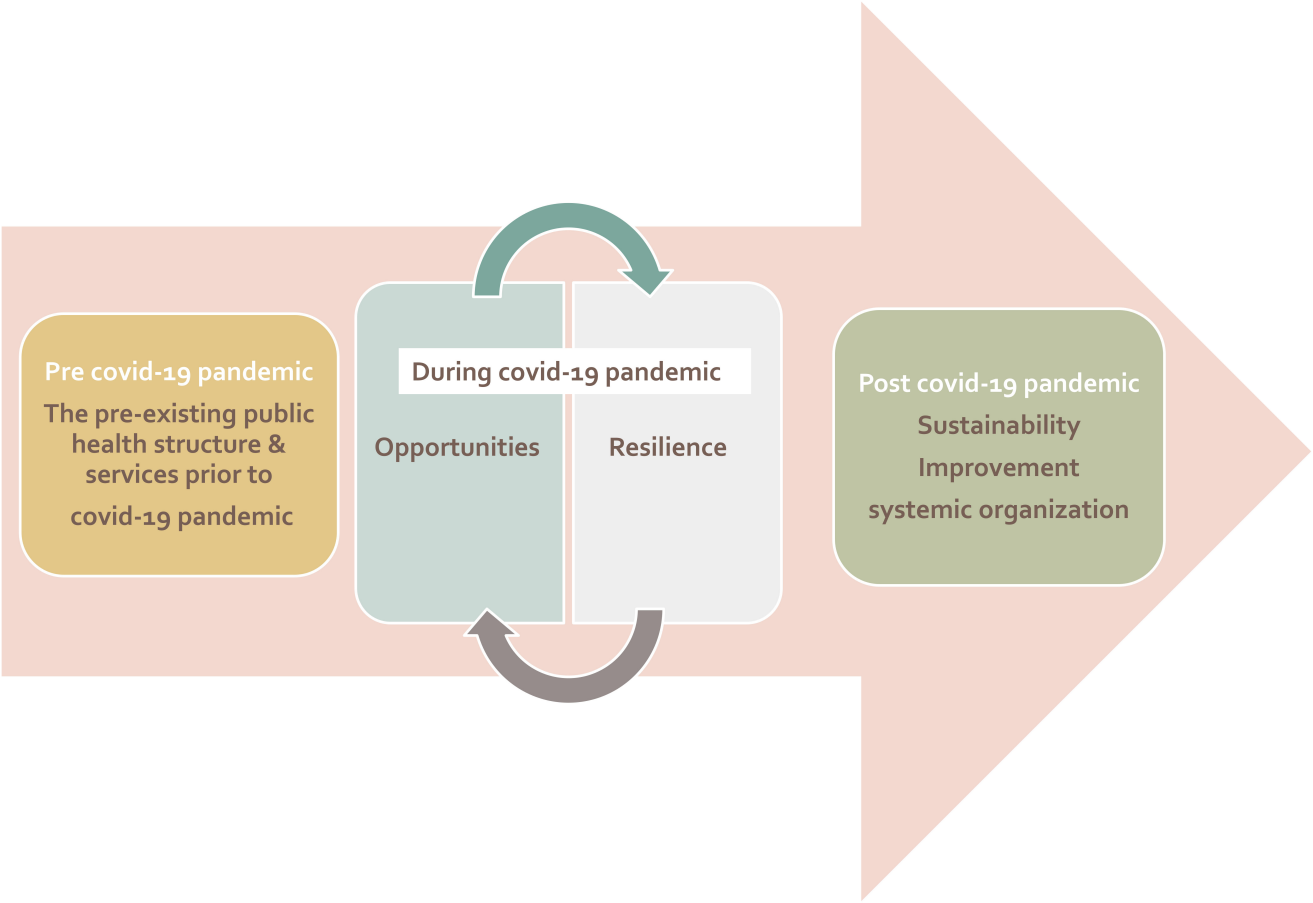
Framework scheme of the review.

The framework of this review is designed to cover three periods in relation to the COVID-19 pandemic: before, during and the desired way forward in post-pandemic. This framework highlights the opportunities generated by the COVID-19 pandemic in the field of public health in Oman (Figure 1).

## Pre-COVID-19: Oman's Geodemographic and Public Health Structure and Services

There were many challenges encountered in Oman during the combating of the COVID-19 pandemic that generated opportunities for improvement (Supplementary Material 1). Oman, with its unique location in the Middle East, shares land borders with three countries, and is connected closely to other countries by sea and air travel. These connections serve social, commercial, religious, touristic, and educational purposes. The diversity of Oman's geography with its many border crossings, and the collectivist culture of large Omani families and extended social connections, create an environment with a high risk of importing the disease and increased risk of transmission within the community. Furthermore, the migrant working class with their need for medical care along with an underdeveloped health insurance system have posed difficulties, including establishing free testing and clinical care and vaccination during COVID-19, to achieve universal health coverage. Consequently, during the COVID-19 epidemic, outbreaks in high-density communal residences of migrant workers (dormitories) largely from the Indian subcontinent were challenging to control, not unlike settings in Singapore (8).

Health care services in Oman are widely distributed and mainly led and financially supported by the government in collaboration with the private sector. At a national level, the Directorate General for Disease Surveillance and Control (DGDSC) is considered the responsible body to run and monitor day-to-day public health work as well as during emergent events and public health crises via its different departments including surveillance, communicable diseases, central public health laboratories, infection prevention and control, environmental, port health and International Health Regulations, and occupational health. However, the DGDSC has no functional arm by itself except through the directorates general for health services in the different governorates. The functional arm which implements and runs the clinical as well as the public health services are the primary, secondary, and tertiary health care facilities, private health establishments and other non-Ministry of Health (MoH) health care facilities.

The scattered structure of public health services with limited resources for detection, response and prevention, necessitated a huge effort to mobilize and train health care workers for the implementation of public health interventions during the pandemic. Additionally, maintaining essential health services, especially during surge periods, was a leading challenge during the pandemic. For example, the primary health care staffs were utilized in COVID-19 response at community level as well as in acute care services at the hospitals.

**Figure 2:**
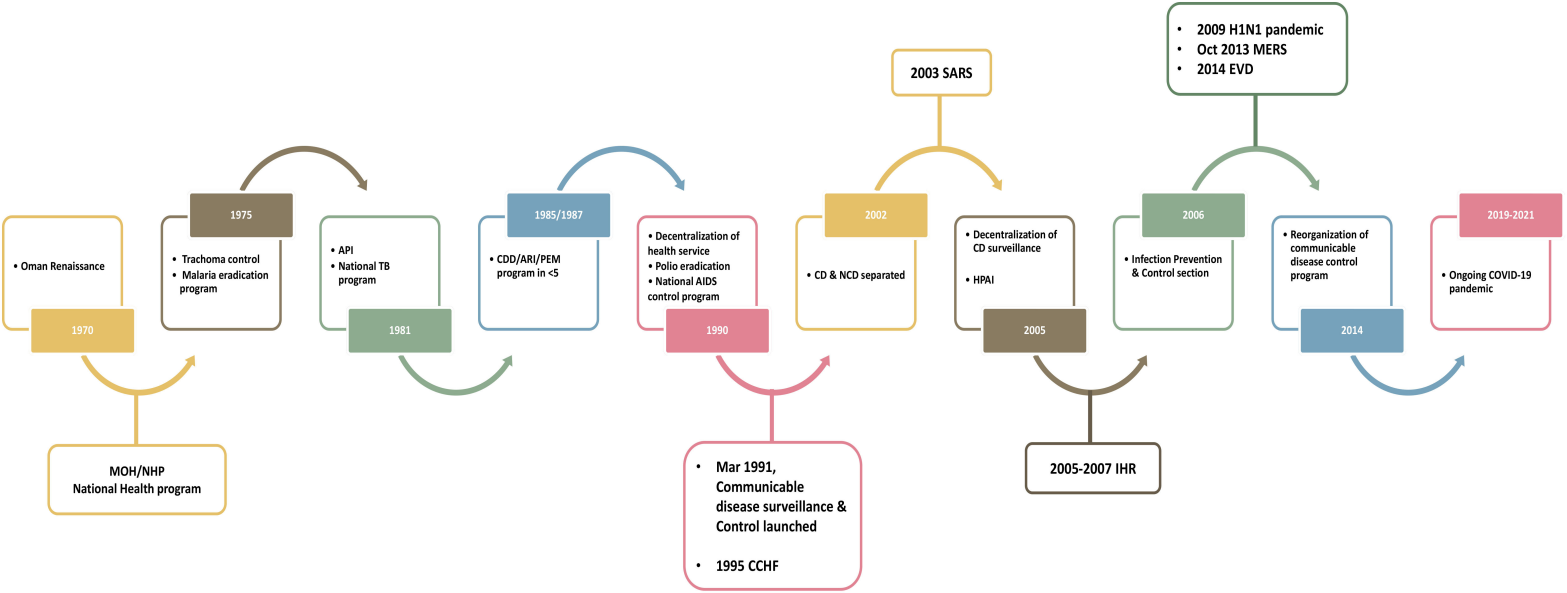
Historical milestone of the public health services and communicable disease control in Oman.

Over the years, Oman experienced different outbreaks such as Crimean-Congo hemorrhagic fever, dengue fever, measles and Middle East respiratory syndrome coronavirus (MERS-CoV) (9–11) (Figure 2). Although Ebola did not evolve into a pandemic in 2014, the preparedness experience for it at Oman's PoEs and in building infection prevention and control capacities was of immense value thereafter (12). Ebola preparedness allowed the involvement of public health in designing the country's new international airport in Muscat with a health care facility linked to the arrival pathway before immigration with six airborne isolation rooms and an exit pathway away from passengers to avoid exposures from potential infectious cases. The management of dead bodies from infectious cases was addressed as a public health hazard during Ebola and MERS-CoV outbreaks where infection control policy was brought forward through scientific risk assessment with religious approval (12). The experience with malaria, CCHF, MERS-CoV, and the later importation of dengue triggered multisectoral collaboration with other sectors–animal health, environment authority, and municipalities–through a committee for integrated vector management and zoonotic diseases. Previous experience from management of local and international threats as well as the JEE were useful in showing Oman's unique capabilities and weaknesses. However, these events have proven the effectiveness in management of short-term crises, but they are limited when it comes to long-term crises such as the COVID-19 pandemic.

For surveillance and reporting of notifiable diseases, an electronic surveillance system (Trassud) was established in 2017 but was only accessible to MoH health institutions. In addition, a national incident command chain existed prior to the COVID-19 pandemic to ensure abrupt reporting and response to acute public health events (Supplementary Material 2). Nevertheless, there was an unclear implementation framework and responsibilities despite the presence and involvement of different stakeholders.

## During the COVID-19 Pandemic: Challenges, Opportunities, and Resilience

Preparedness for the COVID-19 pandemic in Oman started with the announcement of initial cases of SARS-CoV-2 infection in Wuhan in December 2019. For the DGDSC team, this was regarded as a significant public health threat and the existing national incident command chain was activated. Pandemic management was based on risk assessment at national and district levels during the various stages of transmission using the existing all hazard plan, setting guidelines and preparation plans, conducting risk assessments at PoEs, and making field visits to PoEs and health care institutions.

There were daily virtual meetings with all public health officers from each governorate in the country for the first 7 months of the pandemic, until July 2020, and then twice weekly until December 2020, currently, meetings occur on a weekly basis. Discussion included the day-to-day situation analysis of the pandemic and troubleshooting, any new national guidelines or policies, the global situation and travel related issues, strategic plans, and implementation approaches.

Capacities increased to fulfill the needs for detection, response and prevention as described below while observing and maintaining other essential health care services. For example, the primary health care visits in Muscat governorate came down from 115,324 in January 2020 to 109,719 in March 2020 when the epidemic started but the essential health services were ensured for vulnerable groups, women and children (13). During the phase of increase in capacity, several challenges surfaced. This included the lack of infrastructure and trained human resources that played a major challenge in detection, management, contact tracing, and vaccination. Furthermore, the re-organization of health care workers between institutes as well as attitudes and practice toward intervention implementation processes were additional challenges. Moreover, there was a lack of operational plans, governance, and technicality at the workplace due to lack of occupational health experts in institutes including health care institutes.

### Detect

Despite limited human resources, poor logistics, and the scarcity of laboratory tests due to the increased global demand at the start of the pandemic, the central public health laboratory (CPHL) managed to provide the country with the national base of pandemic data. At the beginning of the pandemic, there was limited human resources, shortage of experienced staff in molecular microbiology, limited supply of kits and consumables, limited laboratory and storage space that could accommodate a large number of samples and the lack of integration in the electronic lab information system. In order to tackle these challenges, detection services expanded to cover all health care facilities in both government and private sectors that increased the capacity from the originally available two laboratories at the beginning of the pandemic to 44 laboratories countrywide. Reverse transcription polymerase chain reaction (RT-PCR) and point of care PCR rapid testing were provided to all COVID-19 admission health facilities. Later, rapid antigen testing was also introduced in hospitals, primary health care, workplaces, and communities.

The CPHL, as part of DGDSC, played a pivotal role in the decentralization of testing, training of laboratory personnel, evaluation of kits, auditing the newly established laboratories, and providing quality assurance samples to all testing facilities. With the emergence of COVID-19 variants, CPHL began to establish and build capacity for whole genome sequencing and establishment of a fully functioning bioinformatics system. The role of CPHL was upgraded from testing, training, and building the diagnostic capacity of the other laboratories at the beginning of the pandemic to supervising, auditing, validating, and verifying laboratory processes and tools. Since June 2020, the DGDSC expanded the influenza-like-illnesses (ILI) sentinel sites from two to 13 sites nationally and integrated the sentinel surveillance of influenza and SARS-COV-2 in the ILI and severe acute respiratory infections surveillance programs.

### Respond

#### Unified Government Leadership

Despite Oman going through a political transition with the appointment of the new sultan, the incoming government committed to provide full support for the pandemic response. Thus, a Supreme Committee, including all relevant stakeholders from different sectors, was formed to deal with COVID-19 pandemic disease progression in March 2020.^2^ This was a national priority in response to the pandemic threat to harmonize the response and minimize the impact of the pandemic on life, health, and social, and economic aspects.

**Figure 3:**
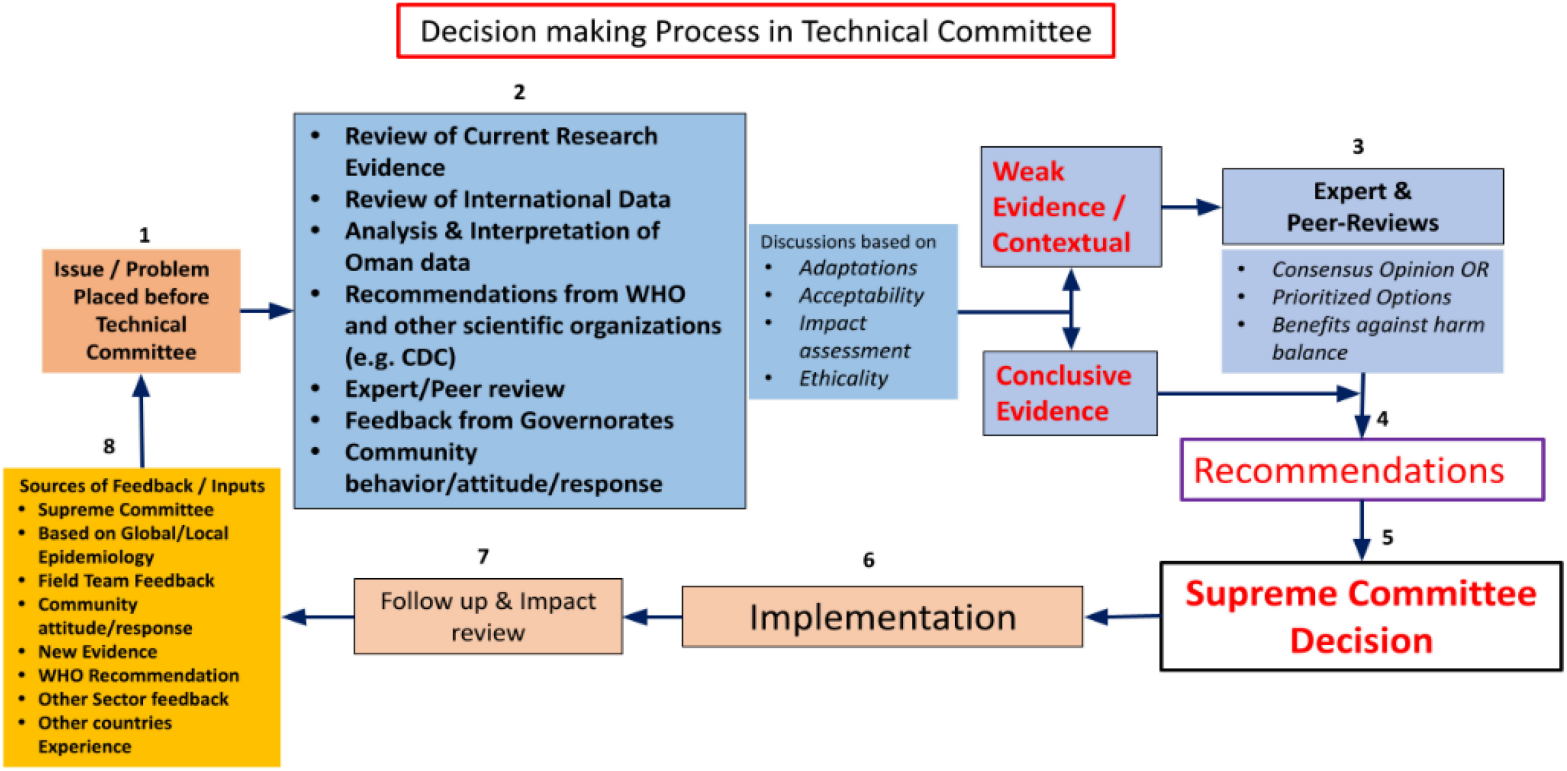
Decision making process during the COVID-19 pandemic in Oman.

The DGDSC was the technical advisory body for the Supreme Committee during the pandemic and oversaw monitoring the national epidemiological data tasked to design models to assess progression of the pandemic that aided decision-making processes of the Committee. The algorithm in Figure 3 illustrates the mechanism of decision making by the technical team.

An example of the work of the technical team is the development of a color-coded alert system (Supplementary Material 3) to monitor the capacity of the health care system for COVID-19 case admission in general and intensive care units based on the peak capacities during different disease waves at national and governorate levels. This was utilized to guide population mobility restriction and other public health measures besides other indicators.

Organizing the hierarchy of mega intersectoral collaboration required during such a pandemic is crucial. As time passes, the lack of governance and legal framework, unclear implementation body and lack of risk communication and infodemic control become clear as one of the major challenges. In addition, establishing a balance between the economic, financial, and social impact of the pandemic while implementing restricted measures remained a primary challenge throughout the pandemic.

#### Upscaling Capacities in Surveillance, Artificial Intelligence (AI), and at PoEs

##### Surveillance

**Figure 4:**
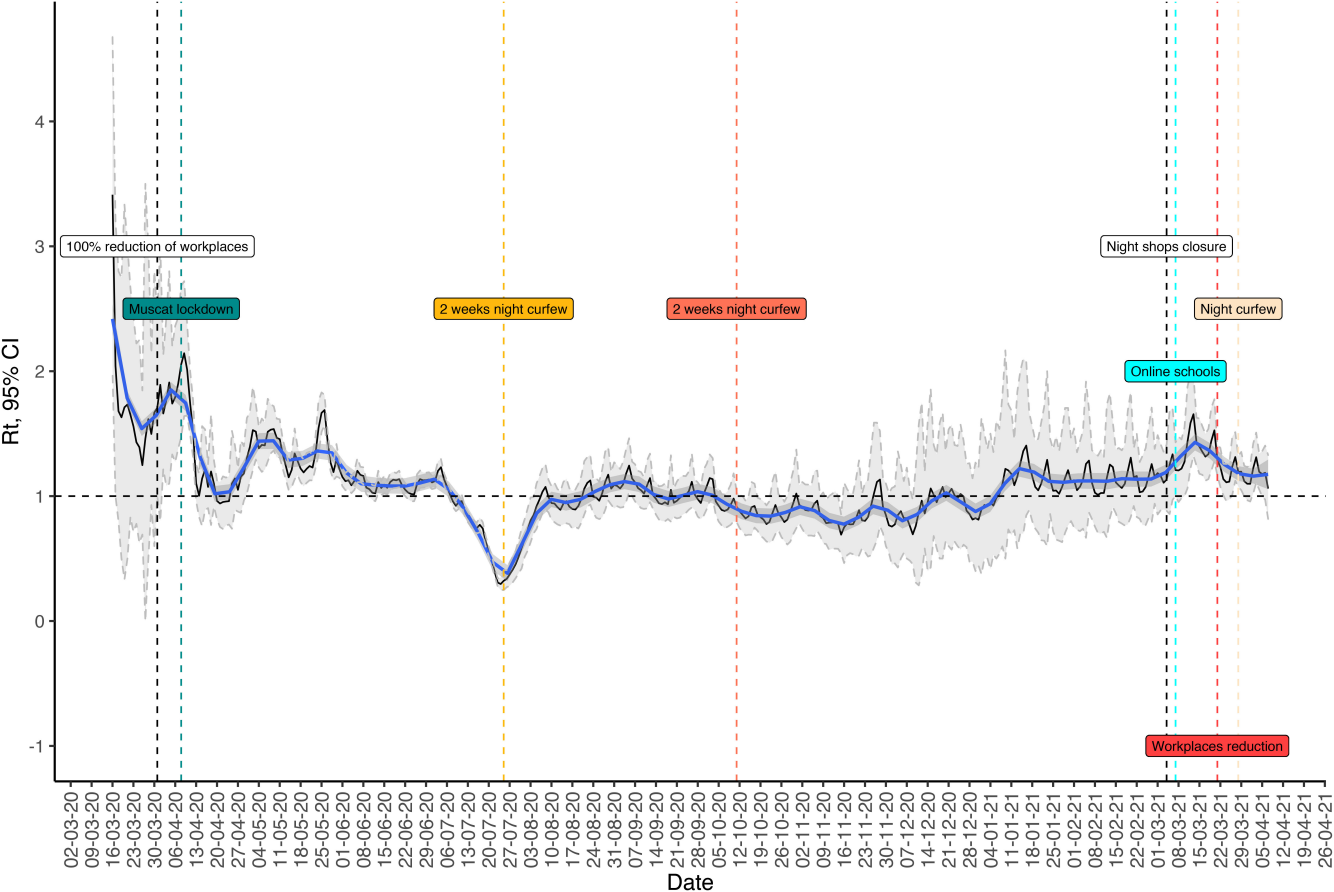
The relationship between the reproduction number and different public health measures during COVID-19 pandemic in Oman.

The Tarassud Plus platform is used as a hub to collect a huge amount of data including all laboratory results for COVID-19 from governmental and non-governmental centers on a daily basis. In this platform, the confirmed, probable and possible cases as well as contacts are digitally monitored. From this, and the monitoring of the daily reproduction number (Rt) curve, which is an indicator of the spread of the disease in the community, the DGDSC generates daily reports on Oman's epidemiological situation (Figure 4). Such monitoring allows for proper evidence-based reports, such as the explanation that the actions taken during the pandemic last year proved effective in reducing Rt (e.g., a 7 p.m. curfew, implementation of the business continuity plan to reduce numbers of non-essential workers at workplaces by at least 70%). The same surveillance system was utilized by the national infection control team to report COVID-19 infection in health care workers and identification of health care associated outbreaks. Nevertheless, monitoring the input from non-governmental institutes and private centers required additional staff and the deficiency of information technology personnel were the main challenges for surveillance.

##### AI

**Figure 5:**
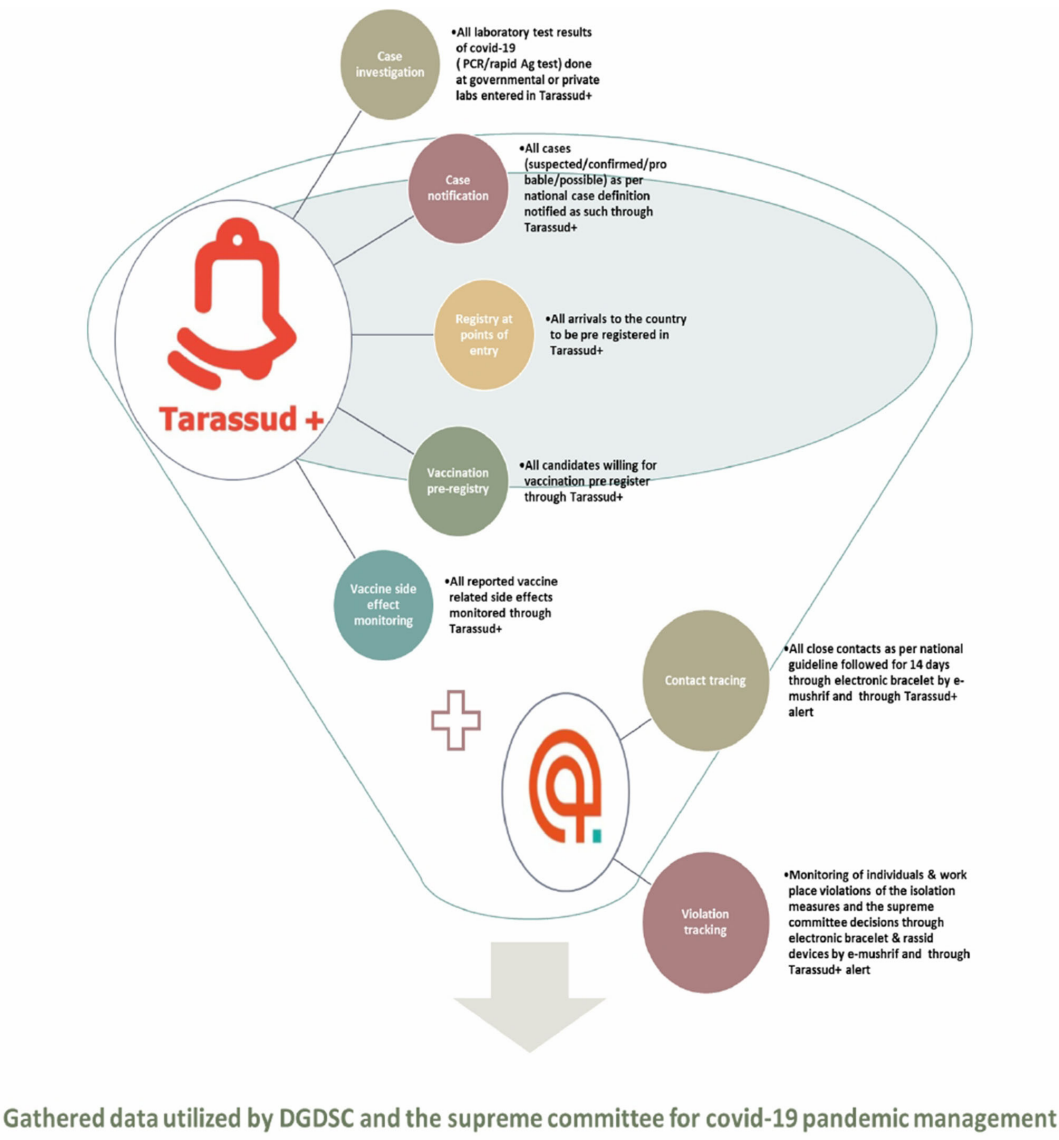
Summary of the role of the Tarassud+ and the e-mushrif during COVID-19 pandemic in Oman.

The role of AI in the COVID-19 response has been evident by expanded utilization of different tools by many countries (14–17). In Oman, AI was introduced early as part of the pandemic response, e.g., surveillance, contact tracing, laboratory testing, and public-private mix. The Tarassud platform has been used to register and monitor travelers in regard to receiving COVID-19 vaccines, track vaccine related adverse events and provide digital health passports (Figure 5). Contact tracing and geofencing in the form of electronic bracelets were used to track confirmed cases, contacts of positive cases and travelers on quarantine. However, as the number of positive cases were growing, sustaining the use of electronic bracelets for contacts was a challenge. Additionally, the availability of good networks and presence of geographical factors, e.g., districts in the mountain, were another challenge in the use of geofencing. High costs of the digital infrastructure and limited funding, the need for around the clock IT support, difficult sustainability, data protection, privacy of personal data, and cybersecurity were some of the challenges in utilization of the AI. Additionally, community acceptance of some AI interventions, e.g., chatbots for risk assessment and follow up, was a challenge. The AI future value as a tool for monitoring other communicable and non-communicable diseases such as individual's compliance with anti- tuberculosis treatment and/or prophylaxis will reduce the pressure on service providers and will be more convenient and time saving for the consumers.

##### PoEs

Controlling and monitoring the travelers at PoEs during the pandemic is a major pillar where preparedness, policymaking, and intersectoral collaboration is established. This includes working with the Civil Aviation Authority, the Ministry of Transportation and Telecommunication, Oman Airports Company, other civil aviation airlines, and land services. Enhancement of public-private mix at PoEs facilitated the establishment of PCR testing facilities at international airports, compulsory pre-registration through Tarassud Plus before arrival to the country and testing on arrival and isolation monitoring through Tarassud Plus. The main challenges at PoEs were the lack of public health services and collaboration between different civil aviation sectors, which were introduced and monitored by different stakeholders during the COVID-19 pandemic. Thus, the operational plan was unclear, and this led to differences in the implementation of required health and safety measures. Moreover, the conflict of interest between stakeholders regarding travel restrictions and its effect on the business was a challenge.

#### Risk Communication and Community Engagement

The participation and mobilization of communities was one of the key components of the national preparedness and response plan. To encourage this, there was collaboration with community organizations such as the Healthy Cities and Villages Initiative, willayat (district) health committees, and community volunteers (18). Awareness activities were implemented at all levels, specifically for infection prevention and control through guidelines that were drafted for places like schools, nurseries, airports, seaports, and dormitories with training and support on implementation and monitoring.

Many efforts were made to raise community awareness and unify media messages to combat COVID-19 including:

- weekly live press conferences of the Supreme Committee were broadcasted live on national TV. The conference updated the public regarding epidemiological situation and any new or change in the national response strategy. The Minister of Health and other members of Supreme Committee addressed questions from public (questions posted in MoH Twitter prior to conference) and media representatives;
- strategic awareness media plans were implemented in collaboration with the Ministry of Information;
- an MoH call center was used to respond to public queries 24/7 throughout the pandemic.

The infodemic at global and national levels has been an additional burden that was reflected in social media as criticism toward the different pandemic interventions. For example, the publics' frustrated response to paying for testing through private sector for travel and other screening purposes, the conflicting opinion regarding home vs. institutional quarantine for return travelers, the demand of lockdown and working from home by employees with stable salaries, and the frustration of businesses from the accompanied financial impact. Addressing the many challenges spurred by the infodemic and open access media was limited at many instances by the high cost of producing media and health promotion materials, the lack of training in risk communication and community engagement, the lack of behavioral scientists who could guide the different approaches to reach the community at different levels of the pandemic.

### Prevent

#### Infection Prevention and Control Capacity

The shortage of personal protective equipment (PPE) was a threat for all healthcare facilities during the COVID-19 pandemic which further stressed frontline health care workers. The involvement of the infection control team in early planning and preparedness at a national level assisted in mitigating this risk and providing safe care throughout the crises by:

- centralizing PPE supply and distribution based on intensity of work and type of care;
- monitoring of utilization and stock of different PPE in all health care facilities on a weekly basis;
- producing risk-based guidelines for use of different types of PPE including N95 respirators;
- providing local supply of PPE through collaboration with local factories and under monitoring of product evaluation team to ensure quality and safe compatibility of products; and
- sourcing medical N95 respirators from firefighters' half face masks

The ongoing communication and feedback between PPE manufacturers, product evaluation teams and frontline health care workers successfully supplied high quality, safe, trusted and user-friendly products even during the stormier periods of the pandemic. The central department for infection prevention and control in collaboration with the directorate of medical supplies at MoH were able to prioritize, mobilize and rapidly respond to shortages, demands or overconsumption. The utilization of private partnerships during the pandemic assisted in extending the airborne isolation facilities and environmental decontamination tools for all referral hospitals in the districts within the country, helping in managing current health care associated transmission risk and building future preparedness of health care facilities for highly communicable diseases. However, the absence of occupational health at workplaces, including in the health care sector, increased the burden on the infection preventionist to cover not only the training of the worker but also the risk assessment and management of exposure in all workplaces at a national level.

#### COVID-19 Vaccination

In 2016, Oman achieved the highest effective vaccine management (EVM) score (99%) for all criteria for all levels out of the 127 EVM assessments conducted globally by World Health Organization and UNICEF in 90 countries by 2016 (19). This experience aided in setting up a national vaccination campaign that aimed to ensure quick delivery of high quality vaccines, through several consultative and negotiating channels through Gavi, the Vaccine Alliance and vaccine manufacturers. Oman has prioritized the categories that must receive the vaccine to those of at-risk groups due to the initial vaccine supply limitation and high demand. Subsequently, a strategy to cover 70% of the total population was set with a deployment plan for administering the vaccine in two phases by priority groups (30% starting in late 2020, then 40% from July to October 2021). This was achieved through an establishment of mega vaccination centers in different governorates with the partnership of the private sector. However, due to the lack of vaccine production, misleading information, and rumors regarding the vaccines, and the inactivation of the vaccine mandate, there are challenges in reaching all the target groups in a timely manner. In addition, due to the difficulty in getting pre-booked quantities of the vaccine from COVID-19 Vaccines Global Access (COVAX), Oman bought the vaccine directly from the vaccine manufacturers at much higher prices than the quantities booked through COVAX. Vaccine hesitancy was tackled through different strategies that included, showing government and community leaders taking vaccine and advocating for it, sharing information about the global and national efficacy of the vaccination *via* different media modalities, showing success examples from other countries for controlling epidemic *via* vaccination, releasing initially daily then weekly vaccine coverage report accompanied by report of the improvement in the national epidemiology situation, and waving testing/quarantine for fully vaccinated asymptomatic individuals as a reward, mandating vaccination to attend public gathering, educational institution, and workplace.

### Operational Research and Collaboration With Different Academic Institutions in Detection, Response, and Prevention

Development in any field can only be achieved by activating the field of scientific research through which decisions are made and reviewed on solid scientific grounds. Examples of research conducted in Oman during the COVID-19 pandemic that aided decision making include a national sero-prevalence survey conducted for COVID-19 disease in the community (20), a multicenter serological study for health care worker exposure risks and infection (21), and a large population-based analysis of severity and mortality determinants (22). Additionally, a pre-campaign cross-sectional knowledge, attitudes, and practices (KAP) study of the COVID-19 vaccine was done (23). These studies aided the selection of priority groups for vaccination. Other research included a molecular epidemiology study for the early transmission of COVID-19 in the country (24), the use of time-varying reproduction number in COVID-19 epidemic monitoring after non-pharmaceutical interventions (25), a study on the impact of mobility restricting interventions during the pandemic (26), and a large population study on the role of children and adolescents in the transmission of the virus (27).

## Lessons and Way Forward in the Post-COVID-19 Pandemic Era

The public health crisis which ensued from the emergence of the SARS-CoV-2 virus resulting in the COVID-19 pandemic has highlighted the vulnerabilities of existing response systems and opportunities for strengthening future preparedness. The response to outbreaks and epidemics is not a new experience for the health care system in Oman but the lengthy duration this pandemic has truly tested the capacity of the current system in sustaining response while observing global development goals. The lack of health insurance for the immigrant workers in Oman, dependence on government funding for health care, the lockdown situations as a restricted mobility intervention for control of the disease spread especially before the availability of vaccines, and the burden of chronic diseases were reflected in the excess mortality rates during 1st year of the pandemic (15%) (28).

This pandemic has not only affected the United Nations Sustainable Development Goal 3 of good health and well-being but also Sustainable Development Goal 4 in the quality of education when the lockdown forced education online without a well-established platform/telecommunication infrastructure especially in the rural areas.

The process of transition to a post-pandemic health care system and building a new era is going to be challenged by grief and exhaustion in individuals and the system itself. The role of public health will project in the recognition and guide high value reflection and empowerment of innovative health care that can withstand future public health threats (29).

The COVID-19 pandemic exposed opportunities for further resource mobilization, capacity building, and communication streamlining to achieve the optimum setting for a multisectoral holistic approach. Opportunities for improvement include the absence of dedicated public health professionals resulting in severe compromises in routine primary health programs, high staff turn-over and limited capacity to run COVID-19 tests at the beginning of the pandemic; inherent gaps in biosafety and security procedures causing challenges in laboratories, the country's multiple points of entry (PoEs) with no dedicated public health personnel to implement screening and quarantine guidelines particularly at the international airport and land crossing; and the lack of inclusivity and monitoring the quality of the private sector health institutions in testing, reporting, tracing, and patient clinical care.

Several lessons taught by the pandemic should not be let go, including the sustainability of what had already been achieved in the field of public health during the COVID-19 pandemic to make the foundation for the establishment of a comprehensive One Health approach at a national level as advocated by recent publications (1). The need for public health funding to meet the essential human capacity, risk communication and expanding implementation of AI is an investment for future national and global security. In the process of transitioning to a One Health approach and implementing value in public health care while minimize destruction, legislation and a monitoring body is crucial to ensure sustainability of local interventions and global coordination. This can be achieved through a formal national public health authority that lead and coordinate all public health services in the country.

As a community case study, this article lacks the assessment tool of the impact of the interventions applied during the COVID-19 pandemic. Additionally, mental, economic, and social effects of the pandemic were not addressed in this article. The line of logic used in the manuscript could have been biased by the personal opinions of the authors.

In conclusion, combating the COVID-19 pandemic is an integrated process. Continuous efforts from all individuals and institutions are essential to reduce its threat. Additionally, there is a need for forward thinking for a public health strategy with empowerment by multiple resources to stand up to public health threats. The transformation of Oman's public health system to take a One Health approach has been informed by syndemic thinking. This approach should continue to be part of the fight against the COVID-19 crises and inform the future vision of a healthy population with a more holistic approach to health, encompassing physical, mental, economic, and social dimensions. A system that works on eliminating health disparities, improving health literacy, and implementing effective communication and dialogue between public health, policy makers and the community is essential to the process of improving public health.

## Data Availability Statement

The original contributions presented in the study are included in the article/Supplementary Material, further inquiries can be directed to the corresponding author.

## Author Contributions

All authors contributed to the writing and editing of the paper and approved the final manuscript.

## Conflict of Interest

The authors declare that the research was conducted in the absence of any commercial or financial relationships that could be construed as a potential conflict of interest.

## Publisher's Note

All claims expressed in this article are solely those of the authors and do not necessarily represent those of their affiliated organizations, or those of the publisher, the editors and the reviewers. Any product that may be evaluated in this article, or claim that may be made by its manufacturer, is not guaranteed or endorsed by the publisher.

## Footnotes

^1^ https://data.gov.om/search?query=Population

^2^ http://omannews.gov.om/Local/ArtMID/416/ArticleID/9985
