# Supplementary figures and images for: Challenges and Opportunities for Public Health Service in Oman From the COVID-19 Pandemic: Learning Lessons for a Better Future
Source: Front Public Health. 2021 Dec 9;9:770946. doi: 10.3389/fpubh.2021.770946 (PMC8695806; doi:10.3389/fpubh.2021.770946)

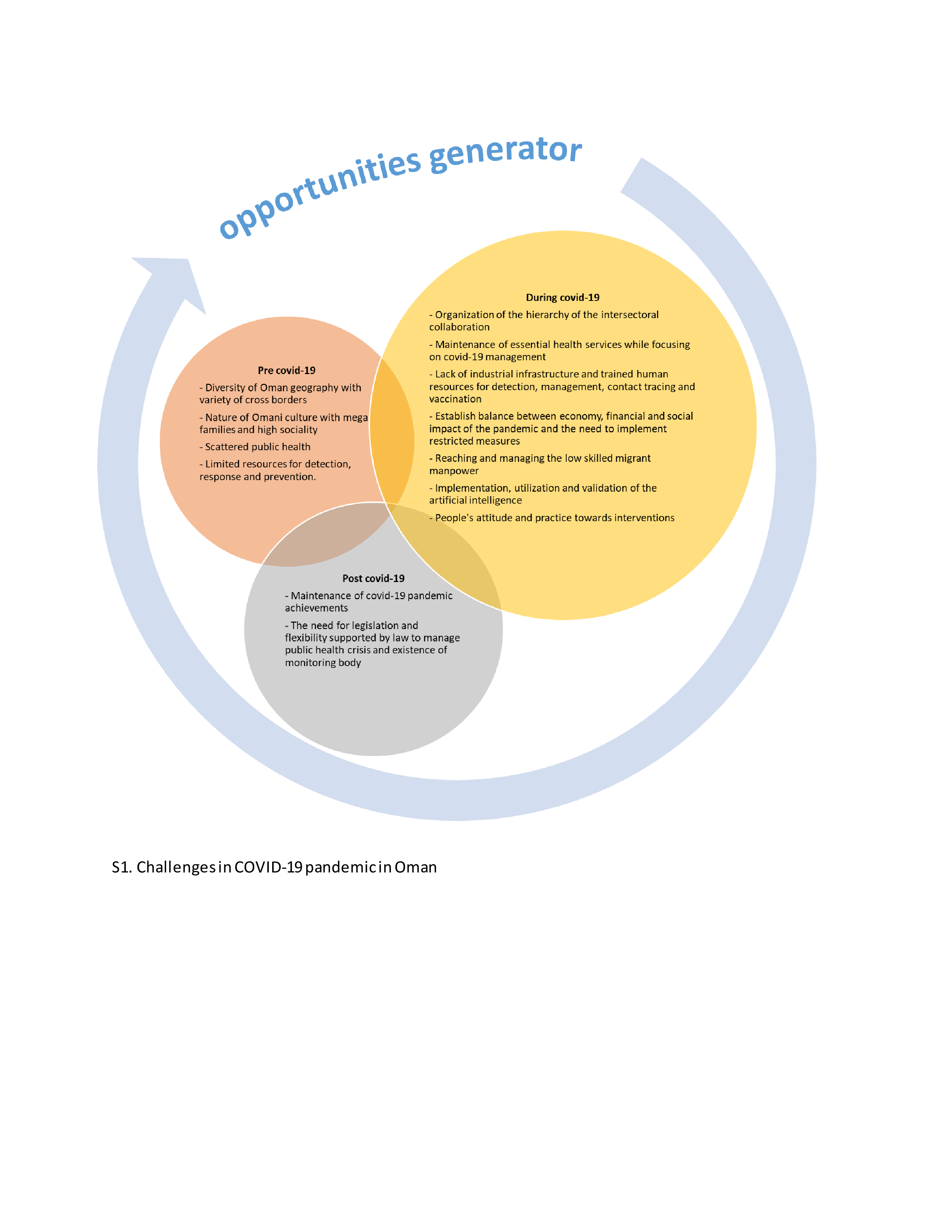

Supplement: Supplementary file 1 [file Image_1.JPEG]

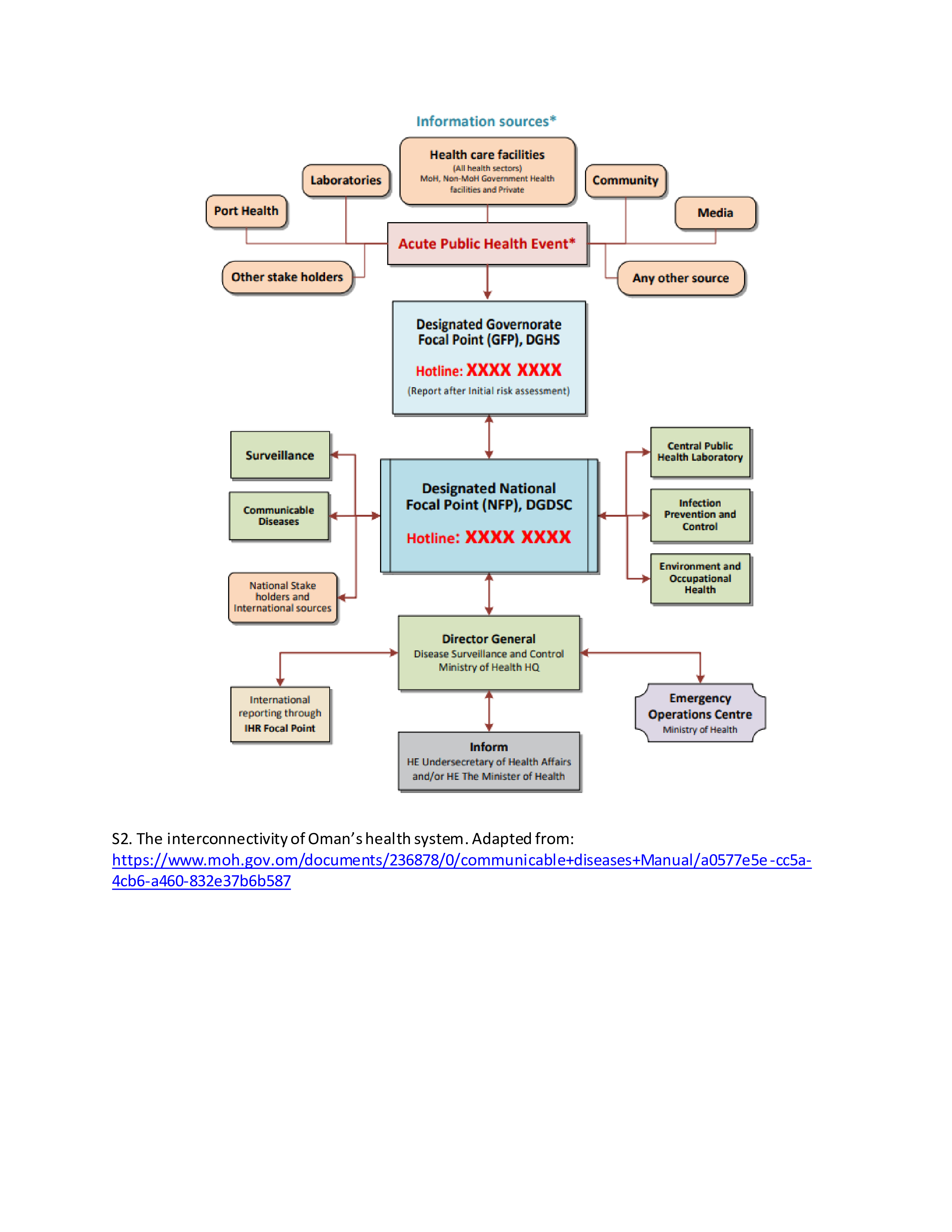

Supplement: Supplementary file 2 [file Image_2.JPEG]

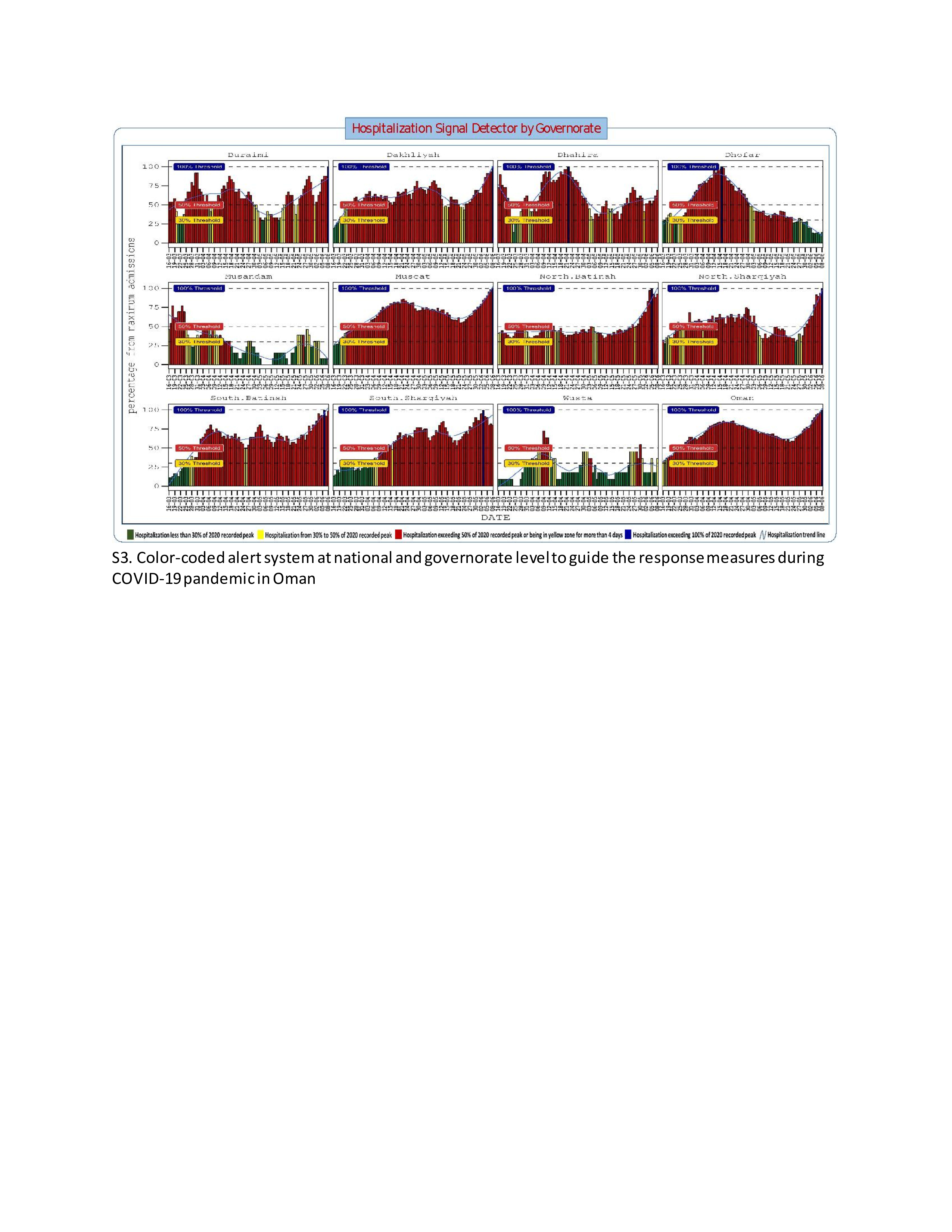

Supplement: Supplementary file 3 [file Image_3.JPEG]
